# Impact of Cork Closures on the Volatile Profile of Sparkling Wines during Bottle Aging

**DOI:** 10.3390/foods11030293

**Published:** 2022-01-22

**Authors:** Filipa Amaro, Joana Almeida, Ana Sofia Oliveira, Isabel Furtado, Maria de Lourdes Bastos, Paula Guedes de Pinho, Joana Pinto

**Affiliations:** 1 Associate Laboratory i4HB—Institute for Health and Bioeconomy, Laboratory of Toxicology, Department of Biological Sciences, Faculty of Pharmacy, University of Porto, Rua Jorge Viterbo Ferreira, 228, 4050-313 Porto, Portugal; famaro@ff.up.pt (F.A.); joana.m.almeida97@gmail.com (J.A.); mlbastos@ff.up.pt (M.d.L.B.); pguedes@ff.up.pt (P.G.d.P.); 2 UCIBIO/REQUIMTE, Laboratory of Toxicology, Department of Biological Sciences, Faculty of Pharmacy, University of Porto, 4050-313 Porto, Portugal; aoliveira@ff.up.pt (A.S.O.); isabel.c.furtado95@gmail.com (I.F.)

**Keywords:** volatile organic compounds, HS-SPME-GC/MS, Italian sparkling wines, cork stoppers, bottle aging

## Abstract

This study aimed at investigating the impact of different technical cork stoppers on the quality preservation and shelf life of sparkling wines. The volatile compositions of two Italian sparkling wines sealed with a sparkling cork with two natural cork discs (2D) and a microagglomerated (MA) cork were determined during bottle aging (12 to 42 months) after disgorging, by headspace solid-phase microextraction coupled with gas chromatography-mass spectrometry (HS-SPME-GC/MS). The volatile profile of the sparkling wine #1 sealed with 2D stoppers showed the presence of camphor from 12 to 42 months, along with significant alterations in the levels of several alcohols, ketones, and ethyl esters at 24 and 42 months. The impact of closure type was less pronounced for sparkling wine #2 which also showed the presence of camphor from 12 to 42 months in 2D samples, and significantly higher levels of esters at 24 and 42 months for 2D compared with MA. These results unveiled that the type of closure has a greater impact on the volatile composition of sparkling wines at longer post-bottling periods and 2D stoppers preserve the fruity and sweety aromas of sparkling wines better after 42 months of bottle storage.

## 1. Introduction

Over the last two decades, the global wine market has experienced an increase in the demand for sparkling wines due to changes in consumers’ preferences [1]. The aroma composition of these wines is a key attribute to consumers’ acceptance and an important indicator of quality [2]. Sparkling wines produced by the traditional method (or *Champenoise*) are relatively complex in terms of aroma composition since they undergo a secondary fermentation in the bottle, followed by different contact periods with lees, when yeasts suffer autolysis releasing nitrogen compounds, polysaccharides, volatile compounds (e.g., ethyl esters), and phenolic compounds, among others (e.g., lipids and nucleic acids) [3], which contribute to the aroma complexity of the greatest sparkling wines.

The impact of several winemaking processes in the aroma composition of sparkling wines has been studied, such as grape variety and maturity [4,5], production methods [6], yeast selection [7], and aging period in contact with lees [7,8]. However, it is well known that the type of bottle closure influences the aroma composition of wines during aging, as has been reported for still wines [9,10], but the aroma comparison between sparkling wines bottled with different types of closures has not been reported so far.

Cork stoppers play a pivotal role in preserving the effervescence (carbon dioxide levels) and the aroma attributes of sparkling wines, making them almost irreplaceable for this type of wine. Nowadays, there are several types of sparkling wine cork stoppers available in the market, from microagglomerated to agglomerated corks, and corks made with agglomerated body plus one, two, or three natural cork discs attached to one of the ends [11]. Importantly, the natural cork discs are obtained from high-quality cork planks, allowing a higher contact of the sparkling wines with this type of material. Hence, this study aimed to investigate, for the first time to our knowledge, the impact of two different technical cork stoppers on the volatile composition of two Italian sparkling wines from 12 to 42 months of aging in bottle after disgorging.

## 2. Materials and Methods

### 2.1. Chemicals

1,4-Cineole (98%), 1-decanol (99.9%), 1-hexanol (99.9%), 1-octanol (≥99%), 2-heptanone (99%), 2-nonanone (97%), 2-undecanone (97%), 3-hexen-1-ol (98%), 5-methyl-2-furfural, benzaldehyde (≥99.5%), camphor (99%), decanal (95%), diethyl succinate (≥99%), ethyl 2-methylbutanoate (99%), ethyl butanoate (99%), ethyl decanoate (≥98%), ethyl heptanoate (≥98%), ethyl hexanoate (99%), ethyl isobutanoate (≥98%), ethyl isovalerate (98%), ethyl nonanoate (≥98%), ethyl octanoate (99%), eucalyptol (99%), furfural (99%), hexyl acetate (98%), isoamyl acetate (≥99%), isoamyl alcohol (98%), limonene (99%), linalool oxide (97%), nonanal (≥95%), octanal (≥98%), phenylacetaldehyde (90%), phenylethyl acetate (99%), phenylethyl alcohol (99%), tartaric acid (≥99.5%), *α*-pinene (99%)), *β*-cyclocitral (90%), *β*-damascenone (≥98%), *α*-ionone (85%), and *β*-linalool (80%) were supplied by Sigma-Aldrich (Madrid, Spain). Ethanol (99.9%) was purchased from ERBA Reagents (Val de Reuil, France).

### 2.2. Sparkling Wine Samples

The sparkling wines used in this study were a 2011 Classic Brut Vintage (sparkling wine #1) and a 2005 Reserve Brut Vintage (sparkling wine #2), from different producers in the Piemonte region in Italy. The sparkling wines were produced from the Chardonnay and Pinot Noir grape varieties, using the traditional method with secondary fermentation in the bottle, and were both disgorged in 2017, corresponding to approximately 5 years of aging in contact with lees for sparkling wine #1 and 11 years for sparkling wine #2. Two types of commercially available stoppers were used for bottling of the two sparkling wines, namely one sparkling cork (3–7 mm diameter granules) with two natural cork discs glued at one end, termed as 2D throughout the article, and one microagglomerated cork (1–3 mm diameter granules), termed as MA. After bottling, all samples were kept under controlled temperature conditions in the producers’ cellars. Samples were then collected at 12, 24, and 42 months (*n* = 3–5 bottles per sampling point) for analysis of the volatile fraction.

### 2.3. Analysis of Volatile Composition by HS-SPME-GC/MS

The analyses of volatile compounds in sparkling wine samples were performed in 2018 (12 months), 2019 (24 months) and 2020 (42 months) using a HS-SPME-GC/MS method adapted from Barros et al. [12]. Briefly, each sparkling wine sample (250 µL) was placed in a 20 mL glass vial which was incubated for 5 min at 45 °C, using a Combi-PAL autosampler (Varian Pal Autosampler, Zwingen, Switzerland). The volatile compounds were then extracted by a 50/30 μm divinylbenzene/carboxen/polydimethylsiloxane (DVB/CAR/PDMS) fiber (Supelco Inc., Bellefonte, PA, USA) for 30 min at 45 °C with a stirring speed of 250 rpm. After extraction, the compounds were thermally desorbed into the GC system for 6 min at 250 °C. All samples were randomly injected.

A 436-GC model (Bruker Daltonics, Bremen, Germany) coupled to a SCION single quadrupole (SQ) mass spectrometer (Bruker Daltonics, Bremen, Germany) and a Bruker Daltonics MS workstation (version 8.2.1, Bruker Daltonics, Bremen, Germany) were used for volatile analysis and quantification. The GC system was equipped with a fused silica capillary column (Rxi-5Sil MS, 30 m × 0.25 mm internal diameter × 0.25 μm; Restek Corporation, Bellefonte, PA, USA) and high-purity helium C-60 (Gasin, Leça da Palmeira, Portugal) was used as carrier gas at a constant flow rate of 1.0 mL/min. The oven temperature was programmed at 40 °C for 1 min, followed by an increase of 5.0 °C/min to 250 °C, where it was held for 5 min, and then increased at 5.0 °C/min to 300 °C. SQ-MS was conducted in the electron ionization (EI) mode at 70 eV and the transfer line, ion source, and manifold temperatures were maintained at 250, 250, 260, and 41 °C, respectively. Data acquisition was performed in full scan mode with a mass range of 40 to 400 *m/z* and a 500 ms scan time. 

For the quantification of volatile compounds, standard compounds were dissolved in a wine model solution (12% ethanol, 5 g/L of tartaric acid, pH 3.2) and analyzed under the same conditions by HS-SPME/GC-MS. The calibration curves were achieved by injecting a range of known concentrations of each compound and computed by the respective area of the peak versus concentration.

### 2.4. Statistical Analyses

Multiple unpaired *t*-tests were applied to evaluate the differences in the levels of volatile compounds in sparkling wines sealed with 2D compared with MA at each post-bottling time. In addition, ordinary one-way analysis of variance (ANOVA) was computed to assess the differences in volatile concentrations between different post-bottling times. The concentration levels were considered significantly different for *p*-values < 0.05. All statistical analyses were performed using the software GraphPad Prism 9 (version 9.3.0, San Diego, CA, USA).

## 3. Results

Bottle closures can affect the aroma composition of wines during aging by three main factors: (1) the oxygen ingress through the bottle which can lead to wine oxidation and the development of oxidized aromas; (2) the desorption of volatile compounds from closures into wine which can lead to pleasant (e.g., terpenes) or unpleasant (e.g., pyrazines) aromas; and (3) the scalping of volatile compounds present in wine by closures [9]. 

**Figure 1:**
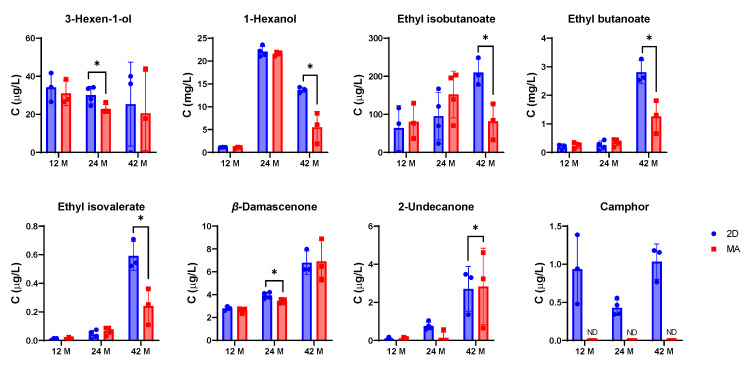
Bar graphs representing the levels of volatile compounds significantly changing in sparkling wine #1 sealed with a sparkling cork with two natural cork discs (2D in blue) and a microagglomerated cork (MA in red) during bottle aging (12 to 42 months). *—*p* ≤ 0.05, ND—not detected.

**Figure 2:**
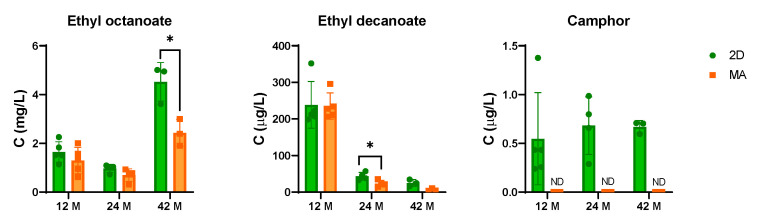
Bar graphs representing the levels of volatile compounds significantly changing in sparkling wine #2 sealed with a sparkling cork with two natural cork discs (2D in green) and a microagglomerated cork (MA in orange) during bottle aging (12 to 42 months). *—*p* ≤ 0.05, ND—not detected.

From the 39 volatile compounds quantified, significant alterations were found for 8 compounds in sparkling wine #1 sealed with 2D compared with MA (Figure 1, Table 1), and 3 compounds for sparkling wine #2 (Figure 2, Table 2), during bottle storage from 12–42 months. Interestingly, a lower number of altered volatile compounds was found for sparkling wine #2 which aged longer in contact with lees (11 years) in contrast with sparkling wine #1 (5 years). The levels of the remaining quantified compounds in both sparkling wines are present in Tables S1 and S2.

The volatile composition of sparkling wines #1 and #2 was more affected by the type of closure at 24- and 42-months post-bottling, while only a qualitative change in camphor (only present in samples sealed with 2D, Figure 1 and Table 1) was detected in both sparkling wines at 12 months. Camphor is responsible for pleasant aromas—such as herbal, minty, and woody [13]—but the olfactory perception threshold in wine or wine model solution has not been reported so far. At 24 months post-bottling, sparkling wine #1 showed significantly higher levels of 3-hexen-1-ol and *β*-damascenone in samples sealed with 2D compared with MA and the presence of camphor only in 2D samples (Figure 1, Table 1). 3-hexen-1-ol is characterized by green and leafy odors and was present in concentrations below the olfactory perception threshold (<400 µg/L) reported for wine model solution [14], while *β*-damascenone is characterized by woody, floral, and herbal odors [14,15], and was present above its olfactory perception threshold (0.05 µg/L) [14]. At 42 months, this sparkling wine showed significantly higher levels of ethyl isobutanoate, ethyl butanoate, and ethyl isovalerate in samples sealed with 2D, as well as significantly lower levels of 2-undecanone and the presence of camphor (Figure 1, Table 1). From these compounds, the three ethyl esters (ethyl isobutanoate, ethyl butanoate, and ethyl isovalerate), and 1-hexanol were present above their olfactory perception thresholds (Table 1) [14], and can contribute with fruity notes [13,15] to the sparkling wine aroma. Interestingly, the levels of 1-hexanol, ethyl butanoate, ethyl isovalerate, 2-undecanone, *β*-damascenone, and camphor changed significantly with bottle aging, while the levels of 3-hexen-1-ol and ethyl isobutanoate were relatively constant over time (Table 1).

**Table 1:** Levels of volatile compounds significantly changing in sparkling wine #1 sealed with a sparkling cork with two natural cork discs (2D) and a microagglomerated cork (MA) during bottle aging (12 to 42 months).

Class/Compound	12 Months ^1^			24 Months ^1^			42 Months ^1^			12 vs. 24 vs. 42 Monthsp	Descriptors ^2^	Olfactory Perception Threshold ^3^
	2D	MA	*p*	2D	MA	*p*	2D	MA	*p*			
Alcohols												
3-Hexen-1-ol (µg/L)	34.2 ± 6.2	31.0 ± 5.3	ns	30.1 ± 4.0	22.8 ± 2.0	*	25.4 ± 18.0	20.6 ± 18.1	ns	ns	Green, leafy	400 μg/L
1-Hexanol (mg/L)	1.1 ± 0.03	1.0 ± 0.1	ns	22.1 ± 1.0	21.6 ± 0.3	ns	13.7 ± 0.5	5.5 ± 2.7	*	****	Green, fruity	8 mg/L
Ethyl esters												
Ethyl isobutanoate (µg/L)	64.0 ± 48.3	80.7 ± 38.0	ns	95.3 ± 54.0	152.3 ± 53.2	ns	210.1 ± 29.0	81.7 ± 38.7	*	ns	Fruity	15 μg/L
Ethyl butanoate (mg/L)	0.17 ± 0.10	0.25 ± 0.09	ns	0.25 ± 0.14	0.35 ± 0.11	ns	2.81 ± 0.33	1.26 ± 0.47	*	****	Fruity, sweet, apple	0.02 mg/L
Ethyl isovalerate (µg/L)	10.0 ± 0.01	13.6 ± 6.4	ns	42.6 ± 24.9	66.8 ± 22.5	ns	591.7 ± 83.5	240.4 ± 102.9	*	****	Fruity, sweet, spice	3 μg/L
Isoprenoids												
*β*-Damascenone (µg/L)	2.79 ± 0.15	2.63 ± 0.19	ns	3.93 ± 0.24	3.42 ± 0.15	*	6.81 ± 0.83	6.92 ± 1.47	ns	****	Woody, floral, herbal	0.05 μg/L
Ketones												
2-Undecanone (µg/L)	0.06 ± 0.08	0.09 ± 0.07	ns	0.76 ± 0.17	0.14 ± 0.25	ns	2.71 ± 0.96	2.83 ± 1.64	*	****	Waxy, fruity	NR
Terpenes												
Camphor (µg/L)	0.94 ± 0.37	ND	Q	0.43 ± 0.09	ND	Q	1.03 ± 0.19	BLOQ	Q	*	Herbal, minty, woody	NR

^1^ Average concentration and standard deviation of sparkling wine #1 sealed with 2D and MA corks. A *n* = 3 per closure was considered at 12 and 42 months, and a *n* = 4 at 24 months. ^2^ Descriptors reported in references [13,15]. ^3^ Olfactory perception thresholds determined in wine model solution as reported in reference [14]. ns—*p* > 0.05, *—*p* ≤ 0.05, ****–*p* ≤ 0.0001, BLOQ–below limit of quantification, ND—not detected, NR–not reported, Q—qualitative alteration.

**Table 2:** Levels of volatile compounds significantly changing in sparkling wine #2 sealed with a sparkling cork with two natural cork discs (2D) and a microagglomerated cork (MA) during bottle aging (12 to 42 months).

Class/Compound	12 Months ^1^			24 Months ^1^			42 Months ^1^			12 vs. 24 vs. 42 Months p	Descriptors ^2^	Olfactory Perception Threshold ^3^
	2D	MA	*p*	2D	MA	*p*	2D	MA	*p*			
Ethyl esters												
Ethyl octanoate (mg/L)	1.65 ± 0.38	1.30 ± 0.48	ns	0.97 ± 0.14	0.70 ± 0.23	ns	4.52 ± 0.65	2.43 ± 0.45	*	****	Fruity, sweet, waxy	0.6 mg/L
Ethyl decanoate (µg/L)	238.5 ± 57.2	235.2 ± 32.1	ns	43.5 ± 9.3	23.6 ± 7.4	*	24.9 ± 7.7	6.3 ± 3.3	ns	****	Fruity, waxy, sweet apple	200 μg/L
Terpenes												
Camphor (µg/L)	0.55 ± 0.42	ND	Q	0.68 ± 0.26	ND	Q	0.67 ± 0.05	ND	Q	ns	Herbal, minty, woody	NR

^1^ Average concentration and standard deviation of sparkling wine #2 sealed with 2D and MA corks. A *n* = 5 per closure was considered at 12 months, a *n* = 4 at 24 months, and a *n* = 3 at 42 months. ^2^ Descriptors reported in references [13,15]. ^3^ Olfactory perception thresholds determined in wine model solution as reported in reference [14]. ns—*p* > 0.05, *—*p* ≤ 0.05, ****—*p* ≤ 0.0001, ND–not detected, NR—not reported, Q—qualitative alteration.

In contrast, at 24 months post-bottling, sparkling wine #2 showed the consistent presence of camphor in samples sealed with 2D, as well as significantly higher levels of ethyl decanoate (Figure 2, Table 2). At 42 months, significantly higher levels of ethyl octanoate and a tendency for higher levels of ethyl decanoate were observed in samples sealed with 2D, along with the presence of camphor (Figure 2, Table 2). The presence of ethyl octanoate in levels above the olfactory perception threshold (>0.6 mg/L) [14] may contribute to the fruity aroma [13,15] of this sparkling wine, while ethyl decanoate may have a lower impact due to its low concentration (<200 µg/L) [14]. Regarding the behavior of these compounds during bottle aging, ethyl octanoate increased significantly, whereas ethyl decanoate showed a significant decrease and camphor levels were constant over time (Table 2).

## 4. Discussion

Ethyl esters are the main class of aroma compounds released by the autolysis of yeasts in sparkling wines produced by the traditional method and they contribute to the fruity and floral-like aromas of these wines [16]. In our study, the levels of several ethyl esters were significantly higher in both sparkling wines sealed with 2D corks. The preservation of ethyl esters during bottle storage has been a challenge for winemakers as ethyl esters tend to hydrolyze over time due mostly to the low pH of wines [17]. Hence, a stopper able to preserve better the ethyl ester composition of sparkling wines can improve their shelf life and the sensory attributes expected by consumers. Notably, most ethyl esters present in both sparkling wines (Table 1, Table 2, Tables S1 and S2)—with exception of ethyl decanoate, hexyl acetate, and phenylethyl acetate—showed a significant increase over time. Despite the behavior of these compounds has been studied during the aging period in contact with lees [7,8], the information about their evolution trends after disgorging is limited [18].

Camphor has been previously identified by our group as only present in wines sealed with natural cork [19], which agrees with the results observed for both sparkling wines sealed with 2D stoppers. The most probable hypothesis is the desorption of camphor from natural cork to wines, which is also corroborated by the detection of this compound in wine model solution extracts of natural cork granules [20]. Based on these facts, camphor seems to be a good marker to discriminate wines bottled with natural cork discs versus other closures.

The levels of two alcohols (3-hexen-1-ol and 1-hexanol), one ketone (2-undecanone) and one isoprenoid (*β*-damascenone) were also significantly influenced by the type of closure in sparkling wine #1. Alcohols can be substrates for wine oxidation originating their correspondent aldehydes [21]. Thus, the lower levels of 3-hexen-1-ol and 1-hexanol in sparkling wine #1 sealed with MA stoppers may be due to their oxidation in hexanal and 3-hexenal, respectively. Though these aldehydes were not detected in the volatile composition of sparkling wine #1 under our experimental conditions. However, 2-undecanone, a ketone that may be also formed by oxidation [21], was found in higher levels in samples sealed with MA. Finally, *β*-damascenone is mainly produced from direct degradation of carotenoid molecules during fermentation [22]. Higher levels of this isoprenoid were previously found in a dry white wine sealed with natural cork compared with microagglomerated cork [23], in agreement with the results obtained for sparkling wine #1 at 24 months.

## 5. Conclusions

These results showed that the type of closure has a greater impact on volatile composition of sparkling wines at longer post-bottling periods (42 months). For both sparkling wines, the sparkling cork with natural cork discs better preserved the fruity and sweety aromas after 42 months of bottle aging, due to the presence of higher amounts of ethyl esters. In addition, the presence of camphor in sparkling wines sealed with a sparkling stopper with natural cork discs seems to be a good marker to discriminate this type of closure versus microagglomerated corks. In general, this work emphasizes the importance of the choice of cork closure for the preservation of the aromatic characteristics of sparkling wines, increasing their shelf life.

## Data Availability

Data available upon request.

## Supplementary Materials

The following supporting information can be downloaded at: https://www.mdpi.com/article/10.3390/foods11030293/s1, Table S1: Concentrations of other volatile organic compounds determined in sparkling wine #1, during bottle aging (12 to 42 months), for which no significant change was observed according to the type of closure.; Table S2: Concentrations of other volatile organic compounds determined in sparkling wine #2, during bottle aging (12 to 42 months), for which no significant change was observed according to the type of closure.
