# Supplementary material for: Impact of Cork Closures on the Volatile Profile of Sparkling Wines during Bottle Aging
Source: Foods. 2022 Jan 22;11(3):293. doi: 10.3390/foods11030293 (PMC8833955; doi:10.3390/foods11030293)
Supplement: Supplementary file 1 [file foods-11-00293-s001.zip › foods-1555628-supplementary.pdf]

Supplementary Material

Impact of cork closures on the volatile profile of sparkling wines during bottle aging

Filipa Amaro [1,2], Joana Almeida [1,2], Ana Sofia Oliveira [2], Isabel Furtado [2], Maria de Lourdes Bastos [1,2], Paula Guedes de Pinho [1,2,*], Joana Pinto [1,2,*]

[1] Associate Laboratory i4HB - Institute for Health and Bioeconomy, Department of Biological Sciences, Laboratory of Toxicology, Faculty of Pharmacy, University of Porto, 4050-313 Porto, Portugal

[2] UCIBIO/REQUIMTE, Department of Biological Sciences, Laboratory of Toxicology, Faculty of Pharmacy, University of Porto, 4050-313 Porto, Portugal

*Corresponding authors:
jipinto@ff.up.pt (J.P)
pguedes@ff.up.pt (P.G.P)

Address:
Associate Laboratory i4HB - Institute for Health and Bioeconomy
Laboratory of Toxicology, Department of Biological Sciences
Faculty of Pharmacy, University of Porto
Rua Jorge Viterbo Ferreira, 228
4050-313 Porto, Portugal
Tel.: +351 220428599; fax: +351 226093390

Table S1. Concentrations of other volatile organic compounds determined in sparkling wine #1, during bottle aging (12 to 42 months), for which no significant change was observed according to the type of closure.

Class/Compound	12 months [1]		24 months [1]		42 months [1]		Units	Descriptors [2]
	2D	MA	2D	MA	2D	MA		
**Alcohols**								
Isoamyl alcohol	188 ± 34	157 ± 41	243 ± 7	252 ± 5	766 ± 68	573 ± 357	mg/L	Banana, burnt cheese
1-Octanol	5.19 ± 0.08	4.79 ± 0.26	4.69 ± 0.30	4.48 ± 0.21	38.1 ± 2.04	34.3 ± 8.48	µg/L	Rose, waxy
Phenylethyl alcohol	53.9 ± 3.8	59.0 ± 12.9	19.3 ± 0.1	19.7 ± 2.2	38.7 ± 10.2	29.2 ± 11.4	mg/L	Rose, honey
1-Decanol	33.2 ± 0.2	21.7 ± 15.4	36.9 ± 9.2	29.4 ± 5.0	26.2 ± 9.7	14.3 ± 10.3	µg/L	Floral, fruity, waxy
**Aldehydes**								
Benzaldehyde	18.3 ± 1.9	19.1 ± 1.9	24.2 ± 2.1	24.2 ± 1.4	24.3 ± 6.8	17.2 ± 9.2	µg/L	Almond, bitter, burnt sugar
Octanal	0.24 ± 0.18	ND	BLOQ	BLOQ	0.78 ± 1.34	1.09 ± 1.44	µg/L	Citrus, fatty
Phenylacetaldehyde	13.8 ± 0.5	13.9 ± 0.3	35.5 ± 2.5	31.6 ± 0.8	86.9 ± 21.8	66.7 ± 21.2	µg/L	Bitter, green, honey
Nonanal	3.73 ± 1.78	1.61 ± 0.06	8.86 ± 4.61	6.68 ± 3.63	30.10 ± 34.2	36.3 ± 31.6	µg/L	Green, pungent
Decanal	8.21 ± 5.70	2.78 ± 1.48	3.68 ± 1.95	1.66 ± 0.47	1.69 ± 0.53	1.54 ± 0.91	µg/L	Grassy, orange
β-Cyclocitral	8.93 ± 0.02	8.92 ± 0.02	0.118 ± 0.204	0.147 ± 0.255	ND	ND	µg/L	Floral, sweet
**Esters**								
Ethyl 2-methylbutanoate	6.55 ± 5.02	9.24 ± 4.25	14.4 ± 10.1	22.2 ± 6.8	30.0 ± 8.2	11.0 ± 8.9	µg/L	Apple
Isoamyl acetate	275 ± 189	376 ± 157	82 ± 42	132 ± 42	623 ± 142	284 ± 191	µg/L	Banana, sweet, fruity
Hexyl acetate	2.18 ± 0.05	1.90 ± 0.27	BLOQ	BLOQ	0.20 ± 0.17	0.11 ± 0.19	µg/L	Cherry, pear
Ethyl hexanoate	0.89 ± 0.15	0.69 ± 0.16	0.49 ± 0.29	0.74 ± 0.22	3.83 ± 1.60	1.60 ± 1.02	mg/L	Apple, banana
Ethyl heptanoate	3.25 ± 0.09	3.35 ± 0.06	0.31 ± 0.19	0.40 ± 0.25	2.73 ± 0.32	2.18 ± 0.45	µg/L	Fruity, melon
Diethyl succinate	14.7 ± 1.5	13.8 ± 1.5	27.9 ± 0.6	28.0 ± 2.1	88.7 ± 15.6	85.6 ± 6.9	mg/L	Fruity
Ethyl octanoate	1.86 ± 0.45	1.16 ± 0.37	0.49 ± 0.24	0.52 ± 0.15	5.43 ± 3.41	3.60 ± 1.55	mg/L	Pineapple, sweet
Phenylethyl acetate	25.4 ± 0.6	23.9 ± 1.0	6.2 ± 0.3	5.9 ± 0.3	4.4 ± 0.25	4.1 ± 0.8	µg/L	Rose, violet

Ethyl nonanoate	9.5 ± 0.4	10.0 ± 0.8	3.5 ± 0.6	3.1 ± 0.8	33.6 ± 7.0	30.3 ± 10.2	µg/L	Rose, fruity
Ethyl decanoate	226 ± 22	204 ± 8	24.5 ± 7.9	13.1 ± 3.3	29.1 ± 20.4	31.7 ± 18.7	µg/L	Brandy, fruity
**Furans**								
Furfural	0.85 ± 0.09	0.76 ± 0.04	1.49 ± 0.18	1.61 ± 0.11	3.70 ± 0.87	3.09 ± 2.14	mg/L	Almond, bread
5-Methyl-2-furfural	43.1 ± 3.9	34.5 ± 3.4	28 ± 2.4	26.4 ± 1.7	42.6 ± 5.4	41.1 ± 11.2	µg/L	Almond, burnt sugar
**Ketones**								
2-Heptanone	0.26 ± 0.08	0.18 ± 0.07	1.93 ± 1.03	2.53 ± 0.51	5.53 ± 2.02	3.09 ± 2.22	µg/L	Sweet, fruity, woody
2-Nonanone	1.75 ± 0.95	2.14 ± 1.05	3.72 ± 1.24	4.06 ± 0.73	3.23 ± 1.35	1.91 ± 1.64	µg/L	Sweet, herbal
**Terpenes**								
α-Pinene	BLOQ	BLOQ	ND	ND	ND	ND	-	Pine
1,4-Cineole	39.3 ± 28.6	64.7 ± 19.6	BLOQ	BLOQ	ND	ND	ng/L	Minty, pine
Limonene	3.03 ± 3.40	0.61 ± 0.20	ND	ND	ND	ND	µg/L	Flowery, green, citrus
Eucalyptol	0.90 ± 1.06	0.09 ± 0.07	BLOQ	BLOQ	ND	0.57 ± 0.99	µg/L	Fresh, mint, eucalyptus
Linalool oxide	238 ± 238	66 ± 12	48 ± 16	42 ± 8	188 ± 132	128 ± 52	µg/L	Citrus, green
β-Linalool	21.8 ± 4.4	18.4 ± 0.2	ND	ND	ND	ND	µg/L	Flower, muscat, lavender
**Norisoprenoids**								
α-Ionone	ND	ND	1.34 ± 0.1	1.20 ± 0.1	2.4 ± 0.3	2.2 ± 0.8	µg/L	Fruity, violet

[1] Average concentration and standard deviation of sparkling wine #1 sealed with a sparkling cork with two natural cork discs (2D) and a microagglomerated cork (MA). A *n*=3 per closure was considered at 12 and 42 months, and a *n*= 4 at 24 months. [2] Descriptors reported in references [21,22]. BLOQ – below limit of quantification, ND – not detected, Q – qualitative alteration.

Table S2. Concentrations of other volatile organic compounds determined in sparkling wine #2, during bottle aging (12 to 42 months), for which no significant change was observed according to the type of closure.

Class/Compounds	12 months [1]		24 months [1]		42 months [1]		Units	Descriptors [2]
	2D	MA	2D	MA	2D	MA		
**Alcohols**								
Isoamyl alcohol	71 ± 12	63 ± 6	252 ± 7	249 ± 11	544 ± 315	489 ± 117	mg/L	Banana, burnt fruit, cheese
3-Hexen-1-ol	ND	ND	22.8 ± 5.4	23.2 ± 2.2	18.7 ± 17.8	9.4 ± 16.2	µg/L	Green, leafy
1-Hexanol	0.26 ± 0.01	0.27 ± 0.01	5.75 ± 0.30	5.53 ± 0.18	1.77 ± 0.99	1.56 ± 0.25	mg/L	Green, fruity
1-Octanol	6.21 ± 0.41	6.43 ± 0.62	6.72 ± 0.67	6.39 ± 1.04	33.6 ± 20.5	34.7 ± 2.9	µg/L	Rose, waxy
Phenylethyl alcohol	70.7 ± 12.2	62.9 ± 5.9	17.9 ± 3.2	19.8 ± 2.1	35.3 ± 20.4	30.3 ± 10.5	mg/L	Rose, honey
1-Decanol	35.4 ± 4.9	33.0 ± 0.3	44.1 ± 13.9	43.3 ± 11.4	9.81 ± 11.6	4.63 ± 6.8	µg/L	Floral, fruity, waxy
**Aldehydes**								
Benzaldehyde	30.4 ± 1.5	32.0 ± 2.7	36.7 ± 4.3	43.9 ± 5.7	25.9 ± 17.4	24.6 ± 3.6	µg/L	Almond, bitter, burnt sugar
Octanal	107 ± 132	77 ± 154	BLOQ	BLOQ	4 ± 6	BLOQ	ng/L	Citrus, fatty
Phenylacetaldehyde	34.4 ± 1.3	33.9 ± 1.7	32.2 ± 8.1	38.9 ± 3.4	89.8 ± 52.6	75.2 ± 21.2	µg/L	Bitter, green, honey
Nonanal	3.69 ± 2.32	4.89 ± 3.86	7.65 ± 7.09	14.3 ± 8.0	6.66 ± 7.76	6.45 ± 11.2	µg/L	Green, pungent
Decanal	1.61 ± 0.47	1.79 ± 1.02	2.20 ± 1.63	3.45 ± 0.73	2.11 ± 0.51	1.84 ± 1.00	µg/L	Grassy, orange
β-Cyclocitral	8.95 ± 0.01	8.95 ± 0.01	0.32 ± 0.32	0.19 ± 0.33	ND	ND	µg/L	Floral, sweet
**Esters**								
Ethyl isobutanoate	113 ± 27	104 ± 13	192 ± 35	161 ± 56	122 ± 76	100 ± 19	µg/L	Fruity
Ethyl butanoate	0.44 ± 0.10	0.48 ± 0.05	0.65 ± 0.09	0.61 ± 0.18	2.56 ± 1.49	2.26 ± 0.29	mg/L	Fruity, sweet, apple
Ethyl 2-methylbutanoate	26.1 ± 6.9	25.2 ± 2.0	51.4 ± 9.2	49.3 ± 11.0	32.2 ± 17.6	24.0 ± 8.6	µg/L	Apple
Ethyl isovalerate	43 ± 10	42 ± 5	174 ± 29	144 ± 49	705 ± 393	567 ± 85	µg/L	Fruity, sweet, spice
Isoamyl acetate	102 ± 22	101 ± 11	141 ± 20	118 ± 36	433 ± 265	323 ± 51	µg/L	Banana, sweet, fruity
Hexyl acetate	1.33 ± 0.07	1.38 ± 0.09	BLOQ	BLOQ	0.13 ± 0.23	BLOQ	µg/L	Cherry, pear
Ethyl hexanoate	1.16 ± 0.24	0.91 ± 0.39	1.43 ± 0.21	1.20 ± 0.40	3.80 ± 1.09	3.28 ± 0.56	mg/L	Apple, banana
Ethyl heptanoate	3.58 ± 0.11	3.53 ± 0.07	1.15 ± 0.19	0.84 ± 0.21	2.80 ± 0.65	2.82 ± 0.27	µg/L	Fruity, melon

Diethyl succinate	27.6 ± 2.3	26.9 ± 1.5	33.5 ± 4.0	35.8 ± 1.8	104 ± 22.8	124 ± 5.7	mg/L	Fruity
Phenylethyl acetate	26.3 ± 1.2	26.6 ± 1.8	8.0 ± 0.7	8.0 ± 0.3	5.6 ± 1.7	5.3 ± 0.3	µg/L	Rose, violet
Ethyl nonanoate	6.0 ± 0.3	6.0 ± 0.5	2.0 ± 0.6	1.5 ± 0.4	22.6 ± 14.6	20.9 ± 3.2	µg/L	Rose, fruity
**Furans**								
Furfural	1.77 ± 0.19	1.77 ± 0.24	4.12 ± 0.33	4.25 ± 0.18	4.62 ± 2.78	4.16 ± 0.99	mg/L	Almond, bread
5-Methyl-2-furfural	110 ± 15	96 ± 16	52 ± 4	51 ± 2	52 ± 27	51 ± 2	µg/L	Almond, burnt sugar
**Ketones**								
2-Heptanone	0.34 ± 0.06	0.43 ± 0.10	2.05 ± 0.23	1.81 ± 0.44	3.36 ± 2.01	2.77 ± 1.80	µg/L	Sweet, fruity, woody
2-Nonanone	1.99 ± 0.40	2.90 ± 0.77	3.51 ± 0.26	4.04 ± 1.40	1.01 ± 0.66	1.39 ± 0.35	µg/L	Sweet, herbal
2-Undecanone	0.09 ± 0.04	0.06 ± 0.04	BLOQ	BLOQ	3.98 ± 1.01	1.15 ± 1.56	µg/L	Waxy, fruity
**Terpenes**								
α-Pinene	ND	ND	ND	ND	ND	ND	-	Pine
1,4-Cineole	ND	ND	ND	ND	ND	ND	-	Minty, pine
Limonene	507 ± 107	655 ± 158	ND	ND	ND	ND	ng/L	Flowery, green, citrus
Eucalyptol	55.7 ± 14.4	60.4 ± 41.8	BLOQ	BLOQ	ND	572 ± 991	ng/L	Fresh, mint, eucalyptus
Linalool oxide	49.3 ± 14.1	64.8 ± 17.7	33.2 ± 7.5	44.2 ± 20.3	179.0 ± 93.9	131.0 ± 55.7	µg/L	Citrus, green
β-Linalool	21.8 ± 4.4	18.4 ± 0.2	ND	ND	ND	ND	µg/L	Flower, muscat, lavender
**Norisoprenoids**								
β-Damascenone	2.31 ± 0.03	2.31 ± 0.05	1.53 ± 0.13	1.58 ± 0.13	3.17 ± 0.67	2.82 ± 0.06	µg/L	Woody, floral, herbal
α-Ionone	ND	ND	1.01 ± 0.04	1.13 ± 0.11	1.45 ± 0.67	1.28 ± 0.07	µg/L	Fruity, violet

[1] Average concentration and standard deviation of sparkling wine #2 sealed with sparkling cork stopper with two natural cork discs (2D) and a microagglomerated cork (MA). A *n*=5 per closure was considered at 12 months, a *n*=4 at 24 months, and a *n*=3 at 42 months. [2] Descriptors reported in references [21,22]. BLOQ – below limit of quantification, ND – not detected, Q – qualitative alteration.
